# The Effect of Irrigation Solutions on the Setting Time, Solubility, and pH of Three Types of Premixed Bioceramic-Based Root Canal Sealers

**DOI:** 10.1155/ijod/1995662

**Published:** 2025-08-19

**Authors:** Kitichai Singharat, Ninnita Wongwatanasanti, Warattama Suksaphar, Pakit Tungsawat

**Affiliations:** College of Dental Medicine, Rangsit University, Pathum Thani, Thailand 12000

**Keywords:** bioceramic sealers, irrigation, pH, setting time, solubility

## Abstract

**Introduction:** Calcium silicate- and strontium (Sr) silicate-based sealers are hydraulic materials that require moisture to initiate and complete the setting reaction. This study evaluated the effects of various irrigants on the setting time, solubility, and pH of premixed bioceramic sealers.

**Materials and Methods:** The sealers investigated in this study were CeraSeal (CS), C-Root (CR) SP, and KP-Root (KPR) SP. Setting time was assessed in accordance with ISO 6876:2012 (*N* = 6). For solubility and pH (*N* = 6), sealers were placed in molds and set for 1.5 times the setting time previously determined in the experimental setting time assessment. The free surfaces of the sealers were then exposed to sodium hypochlorite (NaOCl), chlorhexidine (CHX), normal saline, or distilled water (DW) for 1 min. Solubility and pH values were measured at 1, 7, 14, 21, and 28 days.

**Results:** KPR SP showed the highest pH and solubility in CHX and NaOCl. CS exhibited the lowest solubility and shortest setting time, while CR SP had the highest initial solubility and the longest setting time under dry conditions.

**Conclusion:** Irrigating solutions significantly affected the sealers' setting and dissolution behavior. Sr- containing sealers, such as KPR SP and CR SP, showed increased interaction with CHX and NaOCl, highlighting the importance of material composition in clinical performance.

## 1. Introduction

Filling and sealing of the root canal system after cleaning and shaping are crucial for the success and long-term effectiveness of endodontic treatment [1, 2]. Root canal sealers play an important role in sealing the root canal system by entombing residual bacteria, filling canal irregularities, and creating a fluid-tight barrier [3]. This prevents coronal leakage of bacteria or nutrients that could support microbial growth within the root canal system [4–6]. An ideal root canal sealer should form a hermetic seal, maintain dimensional stability or exhibit slight expansion upon setting, and be biocompatible. Additionally, a root canal sealer should not cause discoloration, and it should have a slow setting time, adhere well to dentin walls, be radiopaque, remain insoluble in oral fluids, resist shrinkage, possess antibacterial properties, and be soluble in commonly used solvents [7].

In recent years, sealer-based root canal obturation has gained popularity because of the development of premixed bioceramic sealers, which contain key components such as calcium silicate and strontium (Sr) silicate. Calcium silicate-based sealers (CSBSs) are classified as hydraulic materials that require water to initiate the setting process. Upon hydration, calcium silicates form a calcium silicate hydrate gel (CSH, CaO·SiO·H_2_O) and calcium hydroxide (Ca(OH)_2_). Some CSBS products also contain calcium phosphate, which contributes to improved setting properties [8]. CSBSs are valued for their excellent biocompatibility, strong antimicrobial activity due to high alkalinity, chemical bonding to dentin, minimal shrinkage, and ability to promote hard tissue formation [9].

More recently, Sr-based sealers have been introduced. These sealers primarily consist of two formulations: Sr silicate-based sealers and Sr-doped CSBSs. The Sr silicate-based form is primarily composed of Sr silicate, while the Sr-doped calcium silicate form has calcium silicate as its main component, with the addition of Sr by solid-state sintering [10] and a structure-directing agent [11].

Sr is widely recognized in the medical field for its role in osteoporosis treatment, as it stimulates preosteoblast proliferation while inhibiting osteoclast differentiation [12, 13]. It has remarkable radiopacity because of its high atomic number [14]. Furthermore, Sr plays a crucial role in bone regeneration and has been extensively studied for its beneficial effects on bone metabolism. Research and clinical applications have demonstrated that Sr significantly increases bone formation while suppressing bone resorption [15, 16]. As a result, interest in Sr-containing materials, including Sr-substituted hydroxyapatite (Sr-HA) [17, 18], bioactive glass (Sr-BG) [19], and calcium silicate (Sr-CS) [11, 20], has increased.

Three bioceramic-based sealers with different primary components were selected for this study. CeraSeal (CS) is a calcium silicate-based material composed primarily of tricalcium silicate (20%–30%) and dicalcium silicate (1%–10%). It has a high pH, good flowability, low solubility [21, 22], excellent biocompatibility [23], and the potential to promote periodontal healing [23].

C-Root SP (CR) is a premixed injectable sealer that is similar to iRoot SP, but calcium silicate is replaced by Sr silicate (SrSiO₃). It exhibits low cytotoxicity, excellent biocompatibility, and strong osteogenic potential [24], and it has been shown to reduce inflammation and support periodontal healing [25].

KP-Root SP (KPR) is a ready-to-use premixed bioceramic composed of zirconium oxide, Sr-doped calcium silicates, Ca(OH)_2_, calcium phosphate monobasic, and filler agents. It offers dimensional stability without shrinkage and has excellent physical properties [26].

Premixed CSBSs, such as iRoot SP, EndoSequence, and CS depend on external moisture to trigger a setting reaction [27]; therefore, the presence of moisture and the type of irrigation solution used can influence the setting process [28]. Excessive drying of the root canal should be avoided, as it may prolong the setting time [29]. Typically, sodium hypochlorite (NaOCl) is the primary irrigant used in standard nonsurgical root canal treatment [30]. In nonsurgical root canal retreatment, chlorhexidine (CHX) can also be used [31], whereas in regenerative endodontic treatment, ethylenediaminetetraacetic acid (EDTA) is preferred [32, 33].

To date, some studies have investigated the effects of irrigation solutions on various properties of premixed bioceramic sealers, such as pushout bond strength [34–36], sealer adaptation [28], and sealer penetration into dentinal tubules [37]. Previous studies focused on BioRoot RCS [38, 39], which is not a premixed bioceramic sealer and can completely harden in the absence of external moisture [27]. On the other hand, premixed bioceramic sealers require external water to completely harden. There is no evidence on how irrigation solutions affect the setting time, solubility, and pH of premixed bioceramic-based root canal sealers. Therefore, the purpose of this study was to evaluate the effects of different irrigation solutions on the setting time, solubility, and pH of premixed calcium silicate, Sr silicate, and Sr-doped calcium silicate-based root canal sealers. Accordingly, this study was conducted based on the following research hypothesis: Null hypothesis (H₀): The irrigating solution does not affect the setting time, solubility, or pH of the three types of premixed bioceramic-based root canal sealers.

Alternative hypothesis (H_1_): The irrigating solution affects the setting time, solubility, or pH of the three types of premixed bioceramic-based root canal sealers.

## 2. Materials and Methods

The materials used in this study were CS (Meta Biomed Co., Cheongju, Korea), CR SP (Innovative Bioceramic, Inc., Beijing, China) and KPR SP (Guilin Kevin Peter Technology Co., Ltd., China). The components of each material are listed in Table 1.

This in vitro study was conducted in accordance with ISO 6876:2012 [40] to assess the effects of different irrigation solutions on the setting time, solubility, and pH of the sealers. Each test was performed on six samples per condition (*n* = 6) on the basis of power calculations in G*⁣*^*∗*^ Power 3.1 (effect size *f* = 0.78, *α* = 0.05, power = 0.80).

## 3. Setting Time Assessment

Gypsum molds (10 mm in diameter × 1 mm in height) were used to evaluate the setting time of materials that require moisture for setting. Prior to testing, the samples were divided into five groups as follows: four contact groups, in which the gypsum molds were immersed in the corresponding irrigation solutions, namely, 2.5% NaOCl (M-Dent, Bangkok, Thailand), 2% CHX (M Dent, Bangkok, Thailand), normal saline solution (NSS), and distilled water (DW: positive control), and one noncontact group (negative control), in which the molds were immersed only in DW. All the molds were subsequently incubated at 37°C with 95% relative humidity for 24 h. The sealers were placed into the molds by gently injecting until the material reached the surface level of the mold.

**Figure 1:**
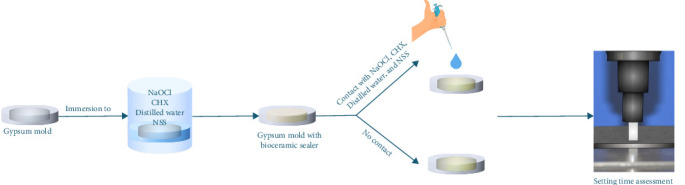
Schematic representation of the setting time assessment.

For each group, 25 µL of the corresponding irrigation solution was applied to the sealer surface using a pipette and gently spread with a sterile plastic inoculation loop. The samples were then incubated at 37°C with 95% relative humidity. The setting time was assessed using a Gilmore indenter with a mass of 100.0 ± 0.5 g and a flat-end diameter of 2.0 ± 0.1 mm, which was vertically lowered onto the horizontal surface of the sample at 10 min intervals during incubation. The sealer was considered set when no visible round indentation was observed. The setting time was recorded from the completion of sealer placement into the molds until the point at which the material had set (Figure 1).

## 4. pH and Solubility Assessment

Solubility was evaluated via the use of metal molds (10 mm in diameter × 2 mm in height), which isolate the bottom and lateral surfaces of the sealer, exposing only the upper surface. The metal mold (*M*) and snap vial (*M*_i_) were weighed to an accuracy of ±0.1 µg to determine their initial masses. The samples were divided into five groups: four contact groups, which involved exposure to 2.5% NaOCl (M-Dent, Bangkok, Thailand), 2% CHX (M Dent, Bangkok, Thailand), NSS, or DW, and one noncontact group with no irrigant exposure. The endodontic sealer was gently injected into the metal molds until it reached the surface level. Subsequently, the mass of each specimen was individually measured.

For the contact groups, 25 µL of the appropriate irrigation solution was applied to the sealer surface using a pipette and evenly spread with a sterile plastic inoculation loop. After 1 minute of contact, the solution was carefully aspirated until no visible moisture remained on the surface of the sealer. All the samples were then incubated at 37°C with 95% relative humidity and allowed to set for a period equivalent to 1.5 times the setting time previously determined in the experimental setting time assessment (*T*₀). After setting, the samples were immersed in 2.717 mL of the designated solutions (immersion ratio ≈ 28.9 mm^2^/µL) and incubated for 1, 7, 14, 21, or 28 days.

The pH of the leachate was measured at each time point using a calibrated pH meter (Sension + PH31; Hach, Loveland, CO, USA) with buffer solutions at pH values of 4, 7, and 14. After each time point, the samples were rinsed with 2 mL of DW, and the rinsing liquid was allowed to drain back into the snap vials. The snap vials were then placed in an oven at 110°C for 24 h to ensure complete evaporation and subsequently reweighed (*m*_f_).

**Figure 2:**
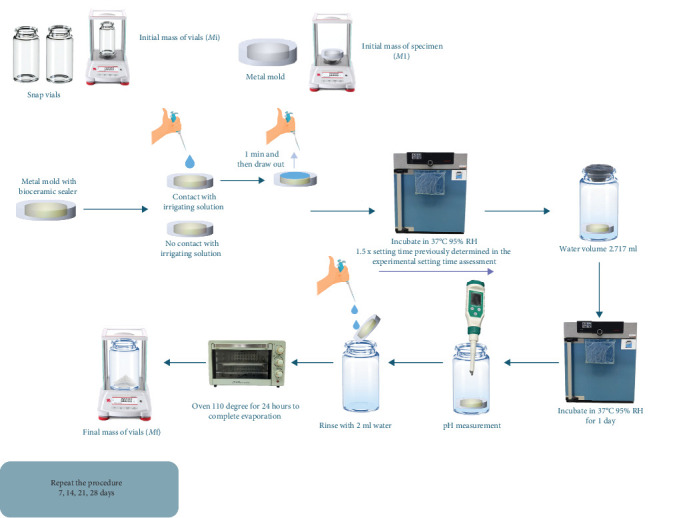
Schematic representation of the solubility and pH assessment.

Solubility was assessed by measuring the mass loss of each set endodontic sealer after immersion in water, in accordance with ISO 6876:2012. A set endodontic sealer should have a solubility of less than 3%. The percentage of mass loss relative to the initial sample weight was calculated and recorded at five-time intervals: 1, 7, 14, 21, and 28 days. Each measurement was performed in triplicate to ensure accuracy and consistency (Figure 2).

## 5. Statistical Analysis

The setting time data were assessed for normality using the Shapiro–Wilk test. For each sealer, differences were analyzed using one-way ANOVA, and pairwise comparisons between sealers were performed using unpaired *t*-tests with *p*-values adjusted using the Benjamini–Hochberg (BH) procedure.

Time-series data for pH and solubility were also tested for normality using the Shapiro–Wilk test. For each sealer, changes across time points (day 1 to 28) were analyzed using one-way repeated measures ANOVA. Pairwise comparisons between time points were conducted using paired *t*-tests, with BH-adjusted *p*-values. All statistical analyses, including descriptive and inferential tests, were performed using SPSS. *p*-Value < 0.05 was considered statistically significant.

## 6. Results

### 6.1. Setting Time

CS exhibited the lowest setting time under dry conditions. Overall, all sealers presented the longest setting times when tested in dry conditions. In the CR group, the shortest setting times were observed with NSS and DW, with values significantly lower than those in the other conditions (*p* < 0.05). Within the CS group, contact with CHX and NaOCl resulted in shorter setting times than dry conditions did (*p* < 0.05). Similarly, KPR displayed the shortest setting time when in contact with NaOCl and NSS, with significant differences from the DW and dry conditions (*p* < 0.05). The setting time data are shown in Table 2.

## 7. Solubility

**Figure 3:**
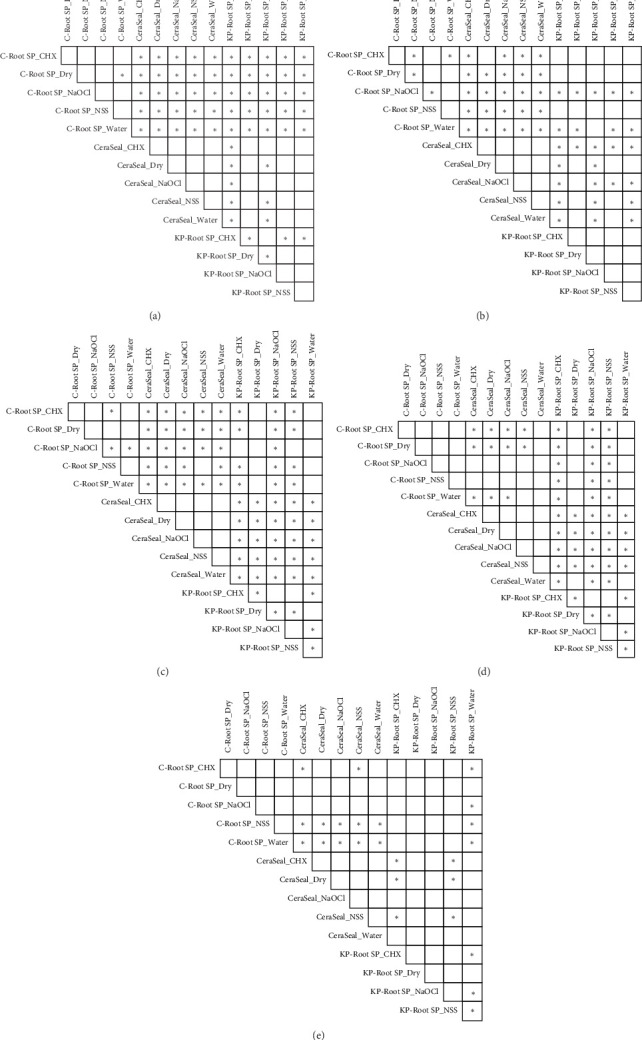
(a–e) Pairwise comparison of the average solubility values among different sealer conditions from the 1st to 28th days. Asterisks indicate significant differences after multiple comparison with the Benjamini–Hochberg (BH) correction. Asterisks indicate significant differences (*p* < 0.05).

On Day 1, CR had the highest solubility compared with the other sealers, with CHX, NaOCl, and water resulting in significantly higher solubilities than NSS and dry conditions (*p* < 0.05). On Days 7, 14, 21, and 28, CR showed no difference in solubility across the irrigation solutions. CS showed no differences in solubility across the irrigation solutions at any time point. At each time point except Day 28, CS had significantly lower solubility than the other sealers. KPR-CHX and NaOCl resulted in significantly higher solubility levels (*p* < 0.05) than did DW and dry conditions across all setting times (Figure 3). The solubility data are shown in Table 3.

## 8. pH

**Figure 4:**
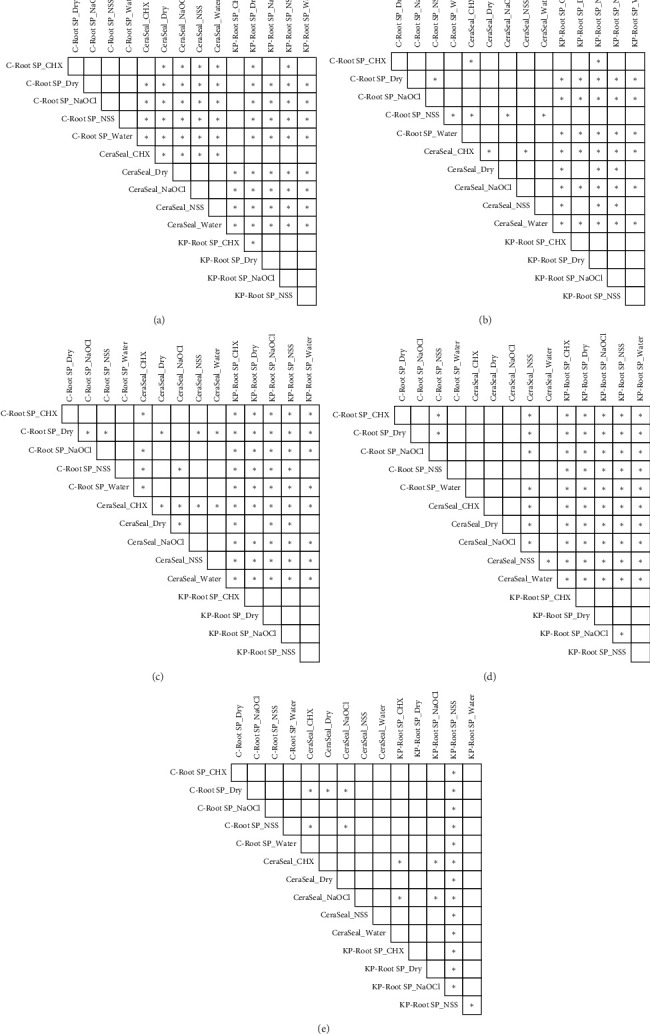
(a–e) Pairwise comparison of the average pH values among different sealer conditions from the 1st to 28th days. Asterisks indicate significant differences after multiple comparison with the Benjamini–Hochberg (BH) correction. Asterisks indicate significant differences (*p* < 0.05).

The pH values of all sealers did not differ between the NaOCl and DW conditions at any time point. Compared with CS and CR, KPR exhibited significantly greater pH values at all time points except Day 28. CR and KPR showed no significant differences in pH across different irrigation solutions at any time point. On Day 1, CR-DW presented the highest pH among all the sealers. On Days 7, 14, and 21, KPR-DW had a higher pH than did CS and CR. On Days 1, 7, and 14, CS-CHX exhibited a significant reduction in pH (Figure 4). The pH data are presented in Table 4.

## 9. Discussion

Bioceramic-based sealers require water to initiate the hydration reaction, which is essential for their setting process and biological properties [39, 41]. Therefore, the dentinal tubules should not be completely dried before obturation [42], as moisture within the canal including any residual final irrigating solution may affect the sealer's physical properties. This study aimed to evaluate the effects of the different irrigating solutions on the setting time, solubility, and pH of premixed calcium silicate, Sr silicate, and Sr-doped calcium silicate-based root canal sealers.

The setting time of bioceramic sealers depends on their chemical composition and moisture conditions [43], which directly affect their properties and the long-term success of endodontic treatment [44]. In the present study, we analyzed sealer setting time via ISO 6876/2012 [40]. Our findings revealed that the setting time of CS under dry conditions was consistent with that reported by Park et al. [45]. Another study that was performed according to ASTM C266-03 and ADA specifications and employed PVC molds reported a prolonged setting time for CS [22].

This study revealed that the setting time of CS was reduced when it was in contact with CHX and NaOCl, which is consistent with previous findings [44] that MTA Fillapex sets faster because of the leaching of heavy metals, such as chromium (Cr), nickel (Ni), and cobalt (Co), which disrupts calcium silicate hydrate crystal formation. In contrast, Kapralos et al. [38] reported that CHX delayed the setting time of BioRoot RCS by binding to phosphate and calcium ions, disrupting hydration and mineralization. Similarly, Jacinto et al. [46]. and Kogan et al. [47]. reported that 2% CHX prevented MTA setting by altering the phosphate level of MTA, which affected the hydration reaction.

Zadsirjan et al. [12] reported that X-ray Diffraction analysis revealed the presence of a tristrontium silicate peak for sealers containing Sr (Sr^2+^), with the intensity of this peak increasing as the Sr^2+^ content increased, while the intensity of the tricalcium silicate peak decreased. This suggests that Sr^2+^ incorporation alters the phase composition of calcium silicate materials [12]. Other studies indicated that the substitution of Sr^2+^ into the calcium silicate structure may interfere with the formation of a hydrated calcium silicate gel, leading to a prolonged setting time [10, 48].

In the present study, CR and KPR presented longer setting times than did CS. Previous research has indicated that the setting time is dependent on the percentage of Sr^2+^. D'Onofrio et al. [49] and Zadsirjan et al. [12] reported that when the Sr^2+^ concentration is less than 25%–30%, the setting time is prolonged. Similarly, Huang et al. [10] demonstrated that incorporating Sr^2+^ into calcium silicate cements delayed both the working and setting times by reducing the precipitation rate of calcium phosphate, as shown by ICP results. Although the mechanism underlying this delayed setting reaction remains unclear, the presence of Sr^2+^ may inhibit the formation of calcium silicate hydrate, resulting in a reduced crystalline phase of calcium silicate after hydration. These findings emphasize the significant impact of Sr^2+^ substitution on the setting reaction of calcium silicate cements, highlighting the need for further research to clarify the precise role of Sr^2+^ in hydration reaction processes.

Our study revealed that contact between KPR and CR sealers and NSS resulted in a shorter setting time. Although no prior research has specifically examined this interaction, the findings can be reasonably explained on the basis of ionic dynamics described in similar studies. According to Radwan et al. [50], the complete dissociation of NaCl in NSS releases Na^+^ and Cl^−^ ions, which can accelerate the hydration of calcium silicate-based materials. It is reasonable to hypothesize that ion exchange may occur between the Sr^2+^ ions present in the sealers and Na^+^ ions from the saline solution. This exchange could increase the availability of Sr^2+^, thereby promoting faster hydration kinetics and reducing the setting time.

In accordance with ISO 6876/2012 [40], sealers that require moisture to set should be tested on a gypsum mold and incubated under moist conditions for 24 h before the setting time evaluation. However, the water absorption of gypsum molds cannot be precisely controlled. Variations in experimental conditions and water content may influence the results, potentially explaining the discrepancies in setting time across studies.

Solubility is determined by measuring the loss of mass of a material after immersion in water. A set endodontic sealer should have a solubility of less than 3%, following ISO 6876/2012 [40]. Higher solubility might lead to compromised long-term sealing ability by creating gaps between the root canal filling material and dentinal walls [43]. On the other hand, the solubility of bioceramic sealers is related to the release of ions, which leads to specific interactions with dentinal walls (mineral infiltration zones) [51] and bioactivity [52].

These three sealers exhibited solubilities exceeding 3% after 24 h of immersion in water at 37°C. Similarly, previous studies have reported high solubility levels for bioceramic materials [21, 53]. The bioactivity of these materials is related to an alkaline pH and the release of Ca; therefore, these materials should be soluble to release bioactive particles [54]. Our results indicated that CS had the lowest solubility among all sealers, with no significant differences observed across solutions at any time point. Similarly, previous studies investigating the impact of root canal irrigation solutions on AH Plus, MTA Fillapex, Bioroot RCS, and PCS reported comparable findings with no effect on solubility [39, 55]. The dissolution of CS is related to the release of Ca ions [22, 56]. Notably, higher solubility in sealers such as CS may increase bioactivity but compromise sealing ability [56, 57]. The optimal balance between solubility and bioactivity requires further study.

CR and KPR exhibited higher solubilities than did CS. According to previous studies, the slightly larger ionic radius of Sr^2+^ than Ca^2+^ may alter the crystalline network, leading to structural degradation and, consequently, increased solubility and ion release in the silicate structure [58]. Similarly, Zadsirijan et al. [12] reported that the addition of Sr silicate enhanced dissolution, as Sr's larger ionic radius than that of calcium led to a less dense and stable structure [59], increasing the susceptibility of the material to dissolution. In addition, a higher Sr^2+^ content further enhances the dissolution effect [12]. Huang et al. [10] demonstrated that incorporating Sr^2+^ into CS cement enhanced degradability by facilitating the release of Sr^2+^ and Si, which affected the ability of the material to stimulate proliferation, osteogenic differentiation, and mineralization. The addition of Sr^2+^ to CS-based 45S5 Bioglass, which is used in bone tissue engineering and regenerative medicine, changes the silicate structure from a pyroxenoid chain to an isolated trisilicate ring. This might cause the Sr–CS cements to dissolve more easily [60].

Our study revealed that exposure to CHX and NaOCl increased the solubility of CR and KPR. Although no previous studies have investigated this topic, a possible explanation is that CHX, a positively charged cationic molecule (26), can bind directly or indirectly to negatively charged regions within the sealer matrix. While Sr^2+^ itself is a positively charged ion, it is not present in isolation [61]; rather, it is embedded within a hydrated matrix that contains negatively charged functional groups, such as deprotonated silanol (Si–O^−^) and hydroxyl (OH^−^) groups [62]. These negatively charged sites may enable electrostatic interaction with CHX, leading to the formation of insoluble CHX─Sr and CHX─Ca precipitates. These precipitates disrupt the internal matrix integrity, increase structural porosity, and consequently increase solubility.

Additionally, NaOCl, with its highly alkaline nature (pH 12–13) [63], promotes rapid alkaline hydrolysis and dissolution of Sr(OH)_2_ and Sr silicate hydrate (Sr─S─H) gels. Its strong alkalinity destabilizes the sealer structure, significantly increasing structural porosity and solubility. Further studies are needed to investigate the molecular basis of these mechanisms.

Bioceramic sealers are hydrophilic and require water for their setting reaction, leading to the production of Ca(OH)_2_ [52]. As a result, solubility testing based on ISO 6876/2012, which measures the elution or washout of water-soluble phases, may overestimate the solubility of hydrophilic sealers [51, 64]. However, high solubility could also be associated with the release of Ca(OH)_2_, which contributes to the sealer's bioactivity [27]. Importantly, the ISO 6876 test does not strictly define solubility, which is based on the thermodynamic equilibrium between a chemical compound and its solution. In reality, the solubility values obtained through ISO 6876 testing may be higher than those observed under clinical conditions [65].

Additionally, studies have shown that the solubility of calcium silicate sealers is significantly lower in synthetic body fluids than in DW [66]. The lower solubility of CSSs in phosphate-containing solutions might be related to mineral precipitation on the sealer surface, a key aspect of bioactivity [67]. Specifically, the presence of phosphate ions allows for the formation of hydroxyapatite and calcium phosphate, suggesting that the in vivo solubility of CSSs is lower than the in vitro solubility. For this reason, revising the methodology for solubility testing of bioceramic sealers may be necessary to ensure more accurate and clinically relevant evaluations.

Alkaline pH plays a crucial role in enhancing the antibacterial activity of a material [68], promoting healing and biocompatibility, supporting osteogenic activity [69], and increasing the deposition of mineralized components [70]. In this study, all the tested sealers were alkaline at all time points; however, the pH gradually decreased over time.

Our results revealed that KPR and CR presented higher pH values than did CS at all time points. This can be explained by Zhu et al. [11], who reported that incorporating Sr into calcium silicate enhances pH stability by reducing the dissolution rate of Ca and Si ions while increasing pH through the release of Sr ions and their interaction with hydroxyl groups. Additionally, Zadsirjan et al. [12] demonstrated that a higher Sr content led to an increased concentration of Sr ions in simulated body fluid (SBF), promoting the formation of Sr hydroxide, which has greater solubility and alkalinity than Ca(OH)_2_ does. These findings are consistent with previous studies, as Sr's larger ionic radius increases solubility and facilitates Sr ion release, contributing to the formation of Sr hydroxide, which is more soluble and alkaline than Ca(OH)_2_ [12, 58].

In this study, CS-CHX significantly reduced the pH, which contrasts with previous research indicating that CHX exposure does not significantly influence the pH of BioRoot RCS throughout the setting period [39]. Since BioRoot RCS utilizes a powder‒liquid mixing system, its setting reaction initiates immediately upon mixing, minimizing the influence of CHX. Conversely, CS is a premixed sealer that depends on moisture within the root canal for setting. During exposure to CHX, this interaction may contribute to the observed pH reduction. This could be due to CHX, a positively charged cationic biguanide molecule that has a strong affinity for divalent cations, such as Ca^2+^ and forms insoluble complexes [71]. This process directly limits Ca(OH)_2_ formation, reducing the release of hydroxyl ions (OH^−^) and leading to a decrease in pH. Prolonged alkalinity of the bioceramic sealer correlated with an increase in solubility [53]. The sealer pH tended to decrease over time, which was correlated with its final setting properties.

In our study, following the method outlined by Kapralos et al. [39], we applied irrigation solutions directly onto the sealers to ensure sufficient contact. Our objective was to use the minimum amount of irrigation solution necessary to adequately cover the sealers' surface area while closely simulating clinical conditions. As described in the materials and methods section, the sealers were immediately exposed to 2% CHX, 2.5% NaOCl, NSS, and DW. The physical properties of the sealers were then evaluated both in the freshly mixed state and at various stages of setting, up to 28 days.

A limitation of this method, as with any in vitro investigation, is the difficulty direct comparison to clinical conditions, where various additional factors can influence the reaction. These include the interaction between the irrigation solution and the sealer, as well as the moisture within the tooth, which plays a crucial role in the setting process.

Further studies are needed to evaluate the influence of irrigation solutions on bioceramic sealers, specifically their surface characteristics and chemical compositions. Optimizing endodontic techniques and materials could ensure that the biological properties of root canal fillings are maintained over time without compromising their physicochemical performance.

## 10. Conclusion

Within the limitations of this in vitro study, irrigation solutions were found to significantly affect the physicochemical properties of premixed bioceramic-based root canal sealers. KPR SP exhibited the highest pH and solubility, particularly following exposure to CHX and NaOCl, whereas CS presented the lowest solubility and the shortest setting times. CR SP exhibited the highest initial solubility and the longest setting time, especially under dry conditions. All sealers presented the longest setting times under dry conditions, emphasizing the essential role of irrigation solutions in the setting reaction. The presence of Sr in certain sealers notably influences their interactions with irrigants, potentially affecting their clinical performance. These findings highlight the importance of selecting irrigants on the basis of sealer composition, and further in vivo studies are recommended to evaluate long-term outcomes and inform clinical protocols.

## Figures and Tables

**Table 1:** The materials used in this study.

Endodontic sealer	Composition
CeraSeal (Meta Biomed Co., Cheongju, Korea)	Zirconium dioxide (45%–50%), tricalcium silicate (20%–30%), dicalcium silicate (1%–10%), tricalcium aluminate (1%–10%), thickening agents and polyethylene glycol [22]
C-root SP (Innovative Bioceramic, Inc., Beijing, China)	Zirconium oxide, strontium silicates, calcium phosphates, calcium hydroxide, tantalum oxide and filler agents
KP-root SP (Guilin Kevin Peter Technology Co., Ltd., China)	Zirconium oxide, strontium-doped calcium silicates, calcium hydroxide, calcium phosphate monobasic and filler agents

**Table 2:** Setting time assessment (mean ± SD; *n* = 6).

Sealer	Setting time (min) (mean ± SD)					Reference group for pairwise *t* test				
	CHX^a^	Dry^b^	NaOCl^c^	NSS^d^	Water^e^	CHX	Dry	NaOCl	NSS	Water
C-root SP	506.33 ± 43.65	605.33 ± 59.66	576 ± 37.84	343.67 ± 8.02	376.5 ± 37.83	*b⁣* ^*∗*^, *c⁣*^*∗*^, d*⁣*^*∗*^ and e*⁣*^*∗*^	a*⁣*^*∗*^, d*⁣*^*∗*^ and e*⁣*^*∗*^	a*⁣*^*∗*^, d*⁣*^*∗*^, and e*⁣*^*∗*^	*a⁣* ^*∗*^, *b⁣*^*∗*^, and *c⁣*^*∗*^	*a⁣* ^*∗*^, *b⁣*^*∗*^, and *c⁣*^*∗*^
CeraSeal	298.33 ± 15.37	352.5 ± 24.24	299 ± 33.99	308.17 ± 37.43	306 ± 45.55	*b⁣* ^*∗*^	a*⁣*^*∗*^ and c*⁣*^*∗*^	*b⁣* ^*∗*^	—	—
KP-root SP	615.33 ± 38.56	664 ± 39.45	587.17 ± 15.11	593.5 ± 40.38	643.17 ± 33.1	*b⁣* ^*∗*^	a*⁣*^*∗*^ and c*⁣*^*∗*^	*b⁣* ^*∗*^ and *e⁣*^*∗*^	b*⁣*^*∗*^ and *e⁣*^*∗*^	c*⁣*^*∗*^ and d*⁣*^*∗*^

*Note: p*-Value was adjusted by the BH correction.

^a^CHX solution and is used for identifying which pairs show significant differences.

^b^Dry condition and is used for identifying which pairs show significant differences.

^c^NaOCl solution and is used for identifying which pairs show significant differences.

^d^NSS solution and is used for identifying which pairs show significant differences.

^e^Water solution and is used for identifying which pairs show significant differences.

*⁣* ^*∗*^ *p* ≤ 0.05.

**Table 3:** Solubility assessment (mean ± SD; *n* = 6).

Sealer	Solution	Solubility (%) (mean ± SD)				
		D1	D7	D14	D21	D28
C-root SP	CHX	54.23 ± 15.13	9.28 ± 4.36	5.34 ± 0.64	2.4 ± 1.11	1.45 ± 1.03
	Dry	48.85 ± 7.95	11.59 ± 5.84	5.03 ± 1.82	2.54 ± 0.94	1.09 ± 0.3
	NaOCl	55.37 ± 4.32	17.97 ± 9.42	6.16 ± 1.56	1.86 ± 0.78	1.22 ± 0.68
	NSS	55.59 ± 5.64	11.45 ± 2.31	3.71 ± 1.22	1.58 ± 1.02	1.63 ± 0.38
	Water	61.7 ± 14.14	16.43 ± 12.84	4.48 ± 0.61	2.13 ± 0.76	1.58 ± 0.44

CeraSeal	CHX	14.75 ± 1.79	1.74 ± 0.95	1.16 ± 0.49	0.77 ± 0.27	0.58 ± 0.34
	Dry	6.75 ± 1.34	3.79 ± 0.88	1.83 ± 0.58	0.93 ± 0.78	0.67 ± 0.32
	NaOCl	14.3 ± 5.84	2.18 ± 0.93	1.8 ± 0.53	0.84 ± 0.65	0.77 ± 0.34
	NSS	6.59 ± 0.7	3.12 ± 0.31	2.6 ± 0.74	1.09 ± 0.45	0.58 ± 0.47
	Water	9.64 ± 2.65	3.05 ± 0.96	1.86 ± 1.19	1.48 ± 0.81	0.8 ± 0.56

KP_root SP	CHX	23 ± 4.83	9.91 ± 1.86	7.15 ± 1.51	4.03 ± 0.98	1.49 ± 0.51
	Dry	8.9 ± 0.58	8.1 ± 0.53	4.98 ± 1.46	2.44 ± 1.18	1 ± 0.51
	NaOCl	18.07 ± 1.25	10.82 ± 1.17	7.79 ± 0.8	4.84 ± 0.96	1.31 ± 0.5
	NSS	11.05 ± 0.77	8.51 ± 0.98	7.06 ± 1.27	4.39 ± 0.93	1.49 ± 0.7
	Water	10.53 ± 0.95	9.51 ± 0.34	4.84 ± 1.9	2.31 ± 0.82	0.36 ± 0.22

**Table 4:** pH assessment (mean ± SD; *n* = 6).

Sealer	Solution	pH (mean ± SD)				
		D1	D7	D14	D21	D28
C-root SP	CHX	12.37 ± 0.11	11.03 ± 0.44	9.63 ± 1.03	8.15 ± 0.31	8.14 ± 0.11
	DRY	12.51 ± 0.05	10.68 ± 1.07	8.98 ± 1.61	8.15 ± 0.2	8.34 ± 0.13
	NaOCl	12.45 ± 0.08	10.78 ± 1.35	10.39 ± 1.1	8.27 ± 0.26	8.18 ± 0.12
	NSS	12.44 ± 0.13	11.69 ± 0.19	10.5 ± 0.44	8.81 ± 0.97	8.26 ± 0.29
	Water	12.47 ± 0.13	10.42 ± 1.08	9.69 ± 1.34	8.28 ± 0.21	8.21 ± 0.05

CeraSeal	CHX	12.2 ± 0.06	9.86 ± 0.99	8.1 ± 0.28	8.2 ± 0.24	7.8 ± 0.14
	DRY	11.63 ± 0.2	10.81 ± 0.54	10.57 ± 0.19	8.39 ± 0.42	7.85 ± 0.1
	NaOCl	11.74 ± 0.44	10.16 ± 0.84	9.53 ± 0.77	8.3 ± 0.27	7.8 ± 0.21
	NSS	11.67 ± 0.19	10.81 ± 0.39	9.99 ± 0.72	9.25 ± 1.09	7.92 ± 0.18
	Water	11.81 ± 0.36	10.14 ± 0.7	10.17 ± 0.5	8.54 ± 0.49	7.92 ± 0.18

KP-root SP	CHX	12.31 ± 0.03	11.94 ± 0.08	11.68 ± 0.14	11.3 ± 0.32	8.25 ± 0.11
	DRY	12.07 ± 0.07	11.71 ± 0.11	11.52 ± 0.17	11.28 ± 0.13	8.2 ± 0.15
	NaOCl	12.21 ± 0.03	11.98 ± 0.02	11.88 ± 0.03	11.4 ± 0.26	8.26 ± 0.2
	NSS	12.13 ± 0.03	11.9 ± 0.04	11.71 ± 0.12	10.67 ± 0.66	9.12 ± 0.92
	Water	12.16 ± 0.09	11.72 ± 0.1	11.39 ± 0.27	10.83 ± 0.42	8.14 ± 0.09

## Data Availability

The datasets generated and analyzed during the current study are available from the corresponding author upon reasonable request.
